# CRISPR/dCas9-Mediated Parkin Inhibition Impairs Mitophagy and Aggravates Apoptosis of Rat Nucleus Pulposus Cells Under Oxidative Stress

**DOI:** 10.3389/fmolb.2021.674632

**Published:** 2021-04-15

**Authors:** Tao Lan, Yu-chen Zheng, Ning-dao Li, Xiao-sheng Chen, Zhe Shen, Bin Yan

**Affiliations:** ^1^Department of Spine Surgery, Shenzhen Second People’s Hospital, The First Affiliated Hospital of Shenzhen University, Shenzhen, China; ^2^Department of Orthopedic Surgery, Shenzhen Luohu People’s Hospital, The Third Affiliated Hospital of Shenzhen University, Shenzhen, China

**Keywords:** CRISPR/dCas9, IDD, mitophagy, apoptosis, Parkin

## Abstract

**Objective:**

The aim of this study is to explore the role of Parkin in intervertebral disk degeneration (IDD) and its mitophagy regulation mechanism.

**Study design and methods:**

Rat nucleus pulposus (NP) cells were stimulated with hydrogen peroxide (H_2_O_2_) to a mimic pathological condition. Apoptosis and mitophagy were assessed by Western blot, terminal deoxynucleotidyl transferase dUTP nick end labeling (TUNEL) assay, and immunofluorescence staining. The CRISPR–dCas9–KRAB system was used to silence the expression of Parkin.

**Result:**

In this study, we found that Parkin was downregulated in rat NP cells under oxidative stress. In addition, treatment with H_2_O_2_ resulted in mitochondrial dysfunction, autophagy inhibition, and a significant increase in the rate of apoptosis of NP cells. Meanwhile, mitophagy inhibition enhanced H_2_O_2_-induced apoptosis. Furthermore, repression of Parkin significantly attenuated mitophagy and exacerbated apoptosis.

**Conclusion:**

These results suggested that Parkin may play a protective role in alleviating the apoptosis of NP cells *via* mitophagy, and that targeting Parkin may provide a promising therapeutic strategy for the prevention of IDD.

## Introduction

Intervertebral disk degeneration (IDD) is widely known as a main contributor to low back pain (LBP), which has become a global public health problem associated with decline in quality of life and heavy socioeconomic burden (Hoy et al., 2010; Foster et al., 2018). It is estimated that approximately 80% of the population suffer from neck or back pain at some point in their lives (Côté et al., 2001). As the largest avascular structure in the human body, the intervertebral disk is a complex structure that consists of superior and inferior cartilage endplates (CEP), internal jelly-like nucleus pulposus (NP), and external annulus fibrosus (AF) (Kadow et al., 2015). NP cells are responsible for the synthesis of the extracellular matrix (ECM) and play an important role in maintaining the biological function of the intervertebral disk. Emerging pieces of evidence reveal that excessive apoptosis of NP cells can trigger IDD (Liu et al., 2013; Chen et al., 2016, 2017). Hence, a better understanding of the apoptosis mechanism may provide a potential therapeutic target for the prevention and treatment of IDD.

Oxidative stress is a common pathological process that is characterized by overproduction of reactive oxygen species (ROS) (Feng et al., 2017). As a main intracellular ROS-generating organelle, mitochondria are also the primary target of ROS. ROS overproduction causes mitochondrial injury. Furthermore, mitochondrial dysfunction enhances ROS generation with a positive feedback loop. Accumulated research has indicated that ROS are a potent pro-apoptotic factor for NP cells (Xia et al., 2019; Xiang et al., 2020; Zhao et al., 2020). Additionally, mitochondria are responsible for the generation of ATP, which is essential for maintaining cell survival and physiological function. Considering that mitochondrial dysfunction is implicated in the senescence and apoptosis of NP cells, mitochondrial homeostasis is vital for the health of intervertebral disks.

Mitophagy is a special type of autophagy that selectively targets damaged or redundant mitochondria to the lysosome for elimination, which is a crucial step in mitochondrial quality control (Novak and Dikic, 2011; Sun et al., 2020). It is acknowledged that mitophagy impairment results in the accumulation of defective organelles and ROS overproduction, and subsequently increases the rate of apoptosis of NP cells (Chen et al., 2020; Kang et al., 2020a, b). Hence, balance between mitophagy and apoptosis determines the fate of NP cells. However, the relationship between mitophagy and apoptosis within disk cells in response to oxidative stress remains poorly understood. Parkin is a key player in the induction of mitophagy. It regulates ubiquitination of mitochondrial outer membrane proteins and promotes degradation of dysfunctional mitochondria. It is reported that Parkin is closely related to the crosstalk between mitophagy and apoptosis in NP cells (Zhang et al., 2018; Huang et al., 2020; Madhu et al., 2020).

In this study, we hypothesized that mitophagy plays a cytoprotective role in response to the oxidative stress of NP cells. To confirm this hypothesis, hydrogen peroxide (H_2_O_2_) was used to induce oxidative stress, which could mimic the pathological mechanisms of mitochondrial dysfunction and apoptosis in NP cells. Furthermore, we investigated the relationship between mitophagy and apoptosis, and the Parkin signaling pathways involved in their interactions. Finally, our study revealed that Parkin is involved in the pathogenesis of IDD and may serve as a therapeutic target for IDD.

## Materials and Methods

### Cell Isolation and Culture

Nucleus pulposus cells were extracted from healthy NP of young Sprague-Dawley rats. NP tissues were isolated under a dissecting microscope and digested in 0.2% type II collagenase for approximately 4 h at 37°C. The isolated cells were cultured in Dulbecco’s modified Eagle medium (DMEM) and 10% fetal bovine serum (FBS) supplemented with antibiotics (Gibco, Carlsbad, CA, United States). Second-generation NP cells were used throughout the experiments.

### Cell Viability Assay and NP Cells Treatment

Cell Counting Kit-8 (CCK-8) assaying was performed to detect the viability of NP cells (CCK-8; Dojindo Co., Kumamoto, Japan) according to the protocol of the manufacturer. NP cells were seeded in 96-well plates and incubated in DMEM/F12 with 10% FBS and 1% antibiotics at 37°C for 24 h. NP cells were treated using H_2_O_2_ with different concentrations (0.1, 0.25, 0.5, and 1 mM) for 24 h or 1 mM for various times (0, 6, 12, and 24 h). The cells were then washed with phosphate-buffered saline (PBS), and a 10 μl CCK-8 solution was added to each well. The wells were incubated at 37°C for 1 h. Finally, the absorbance of the wells was then measured at 450 nm using a micro-plate reader (BioTek, Winooski, VT, United States).

### Quantitative Real-Time PCR (RT-qPCR)

Total RNA was extracted from the cultured cells using a TRIzol reagent (Invitrogen, Carlsbad, CA, United States). Reverse transcription and gene amplification procedures were conducted according to the instructions of the kit manufacturer (TransGen Biotech, Beijing, China). Glyceraldehyde 3-phosphate dehydrogenase (GAPDH) was used to normalize the gene expression of other mRNAs. PCR primers were as follows.

### Western Blotting

The cells were lysed using radioimmunoprecipitation assay (RIPA) buffer (Sigma, St. Louis, MO, United States). Total protein extracts from NP cells were obtained through whole-cell lysis assaying (KeyGen). Protein concentration was determined using the bicinchoninic acid (BCA) method. Protein samples were separated using sodium dodecyl sulfate–polyacrylamide gel electrophoresis (SDS–PAGE) and transferred to a polyvinylidene fluoride (PVDF) membrane. The membrane was incubated with primary antibodies against P62 (1:1,000, ab56416 Abcam, Cambridge, United Kingdom), Parkin (1:1,000, #4211; CST, Danvers, MA, United States), LC3 (1:1,000, #83506, CST, Danvers, MA, United States), Bcl-2 (1:1,000, #3498; CST, Danvers, MA, United States), Bax (1:1,000, #14796; CST, Danvers, MA, United States), GAPDH (1:10,000, #60004-1-Ig: Proteintech) overnight at 4°C; and target protein bands and internal reference bands were visualized and calculated using the ImageJ software (ImageJ 1.48v, United States).

### Immunofluorescence

Nucleus pulposus cells were fixed with 4% paraformaldehyde for 15 min and permeabilized with 0.5% Triton X-100 for 30 min. After blocking with 5% bovine serum albumin (BSA) for 30 min, slides were incubated with primary antibodies against P62 (1:200) overnight at 4°C. Secondary antibody (1:100; Invitrogen, Carlsbad, CA, United States) was added the next day. The nuclei were stained with 4′,6-diamino-2-phenylindole (DAPI) for 1 min. The cells were observed using a fluorescence microscope (CTR4000B, Leica, Wetzlar, Germany).

### Mitochondrial Membrane Potential Measurement, ATP, and Complex III

The mitochondrial membrane potential was determined by JC-1 staining (Beyotime, Shanghai, China) according to the protocol of the manufacturer. Intracellular ATP and complex III levels were determined using the ATP Assay and ROS Assay Kits according to the instructions of the manufacturer (Beyotime, Shanghai, China).

### Flow Cytometry Assay of Apoptosis

The apoptosis rate of the NF cells was detected using the Annexin V-FITC/PI Double-Staining Kit (Fushen, Shanghai, China). NP cells were collected and washed with PBS and subsequently suspended in 100 μl binding buffer. The cells were then incubated with 5 μl Annexin V-FITC and 5 μl PI at 37°C for 30 min. The early (Annexin V+/PI−) and the late apoptotic (Annexin V+/PI−) cells were used to calculate apoptosis rate.

### Transfection of Plasmids

Nucleus pulposus cells were plated using six-well plates overnight before transfection. When the cell density reached 70% confluence, the NP cells were co-transfected with sgRNA and dcas9-KRAB plasmids using Lipofectamine 2000 (Invitrogen, Carlsbad, CA, United States) according to the protocol of the manufacture.

### Statistical Analysis

Results were presented as the mean ± SD and analyzed using SPSS 22 (IBM Corp., Armonk, NY, United States). Differences between groups were analyzed by Student’s *t*-test or one-way analysis of variance (ANOVA) followed by Tukey’s test. A *p* value of less than 0.05 was considered significant.

## Results

### Hydrogen Peroxide Increases Rate of Apoptosis and Inhibits Mitophagy in Rat NP

**FIGURE 1:**
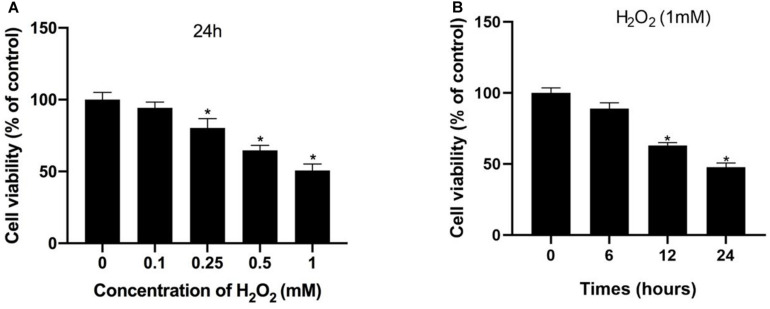
Hydrogen peroxide inhibited cell viability in NP cells. **(A,B)** Effects of H_2_O_2_ on cell viability were detected using the Cell Counting Kit-8 (CCK-8). *p < 0.05 vs. control group.

**FIGURE 2:**
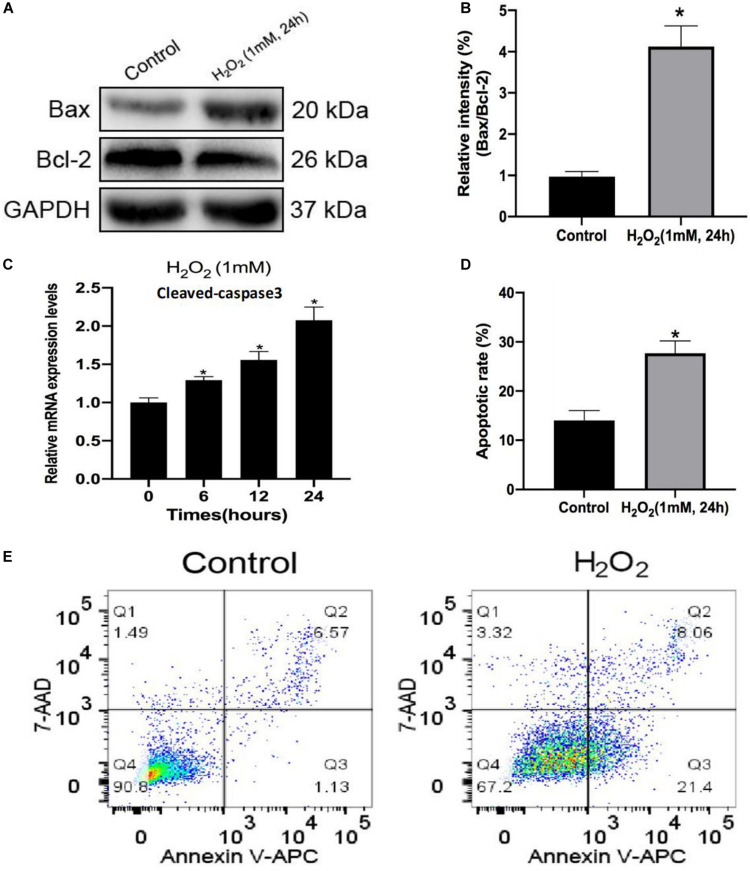
Hydrogen peroxide promotes apoptosis in rat nucleus pulposus (NP) cells. **(A,B)** The protein levels of Bax and Bcl-2 in NP cells were measured by Western blotting. **(C)** Relative mRNA expression of cleaved caspase-3 by RT-qPCR. **(D,E)** Apoptosis of NP cells with or without H_2_O_2_ treatment was detected by flow cytometry. Data are represented as the mean ± SD. *p < 0.05 vs. control group.

**FIGURE 3:**
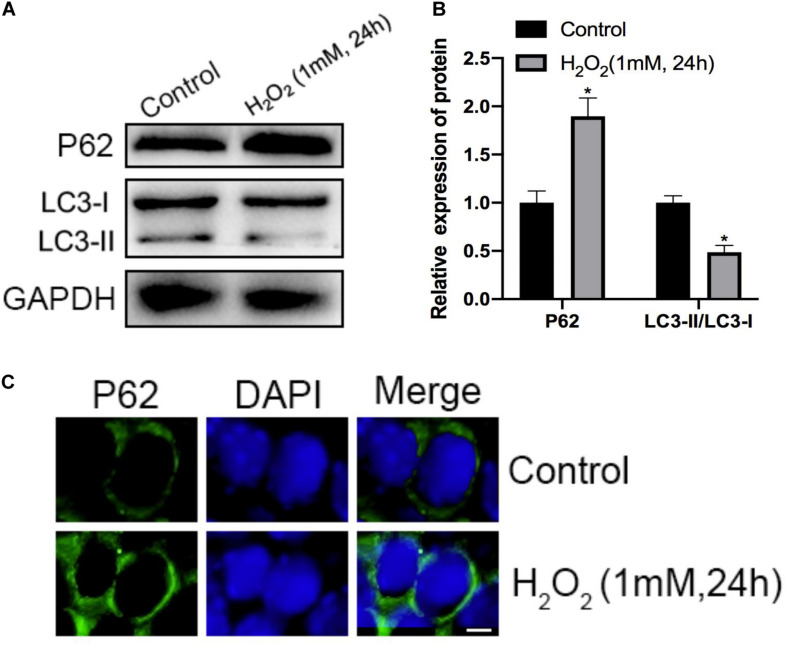
Effects of H_2_O_2_ on mitophagy in NP cells. **(A,B)** The protein level of P62, LC3-I, and LC3-II in the NP cells was measured by Western blotting. **(C)** Immunofluorescence of the P62 protein in NP cells (Green signal represents P62, blue signal represents DAPI). *p < 0.05 vs. control group.

Initially, H_2_O_2_ was used to mimic oxidative stress *in vitro*, and CCK-8 assaying was performed to investigate the cytotoxicity of H_2_O_2_ in NP cells. As shown in Figures 1A,B, H_2_O_2_ treatment was observed to reduce cell viability in a dose- and time-dependent manner. Exposure to H_2_O_2_ with a concentration exceeding 1 mmol/L for 24 h and treatment with H_2_O_2_ (1 mmol/L) for 24 h or longer showed a marked reduction in cell viability. Therefore, H_2_O_2_ with a concentration of 1 mmol/L was used in the subsequent experiments. Then, we assessed the apoptosis response of the NP cells to oxidative stress. The Western blot and qPCR results showed that H_2_O_2_-induced oxidative stress did significantly increase cleaved-caspase3 and Bax/bcl-2 (Figures 2A–C). Meanwhile, flow cytometry by Annexin V-FITC/PI staining showed that the percentage of apoptotic NP cells was higher in the H_2_O_2_-treated group than in the control group (Figures 2D,E). To investigate the effect of H_2_O_2_ on autophagy in the NP cells, we examined the expression of LC3-II/LC3-I and p62 by Western blot. As shown in Figures 3A,B, the ratio of LC3-II/LC3-I was decreased and the level of p62 was increased after H_2_O_2_ treatment for 24 h. The immunofluorescence analysis of NP cells revealed an accumulation of P62 protein in response to oxidative stress (Figure 3C). Taken together, these results indicated that oxidative stress induced by H_2_O_2_ could promote apoptosis and inhibit autophagy in NP cells.

### Mitochondrial Dysfunction Is Involved in the H_2_O_2_-Induced Apoptosis of Rat NP

**FIGURE 4:**
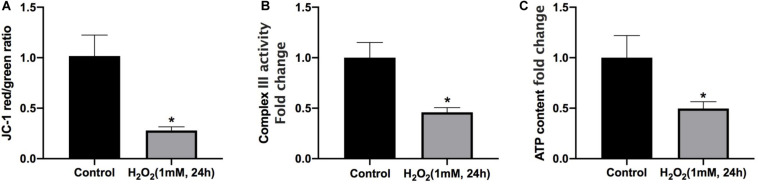
Mitochondrial dysfunction is involved in H_2_O_2_-induced apoptosis of rat NP. **(A)** Effect of H_2_O_2_ on mitochondrial membrane potential. **(B)** Summary data for the effect of H_2_O_2_ on the relative content of complex III. **(C)** Intracellular ATP levels in NP cells. *p < 0.05 vs. control group.

Mitophagy is responsible for quality control of mitochondria under oxidative stress. As expected, oxidative stress inhibits mitophagy but also causes mitochondrial dysfunction. After treatment with H_2_O_2_, the green/red fluorescence ratio of NP cells significantly increased, indicating that mitochondrial membrane potential was reduced by the treatment of H_2_O_2_ (Figure 4A). Furthermore, the intracellular complex III level and ATP production were markedly decreased under oxidative stress (Figures 4B,C). Altogether, prolonged oxidative stress leads to mitochondrial damage.

### Mitophagy Inhibition by 3-MA Enhances H_2_O_2_-Induced Apoptosis in Rat NP

**FIGURE 5:**
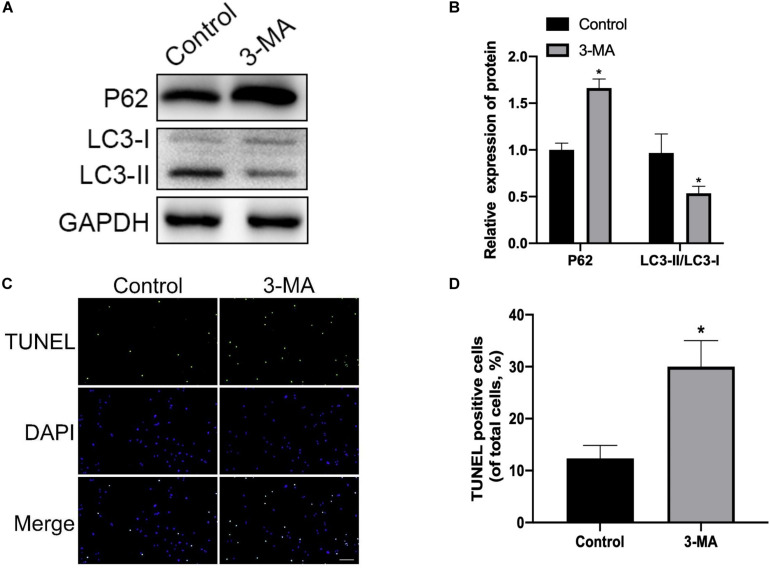
Mitophagy inhibition enhances H_2_O_2_-induced apoptosis in rat NP. **(A,B)** Western blot of P62, LC3-I, and LC3-II expression in NP cells after treatment with 3-MA. **(C,D)** Representative images of TUNEL staining and quantitative analysis showing NP cells apoptosis after treatment with 3-MA. *p < 0.05 vs. control group.

To explore whether mitophagy was involved in the protective response against H_2_O_2_ induced apoptosis, we determined the expression of cleaved-caspase3, Bax, and Bcl-2. We found that NP cells pre-treated with mitophagy inhibitor 3-MA significantly increased pro-apoptotic protein expression (cleaved-caspase3 and Bax) and decreased anti-apoptotic protein (Bcl-2) expression (Figures 5A,B). Similarly, TUNEL assay results also showed that the mitophagy inhibitor dramatically exacerbated the apoptosis of NP cells (Figure 5C,D). Collectively, these findings showed that under oxidative stress mitophagy protected rat NP cells against apoptosis.

### Inhibition of Parkin by dCas9-KRAB Significantly Attenuates H_2_O_2_-Induced Mitophagy

**FIGURE 6:**
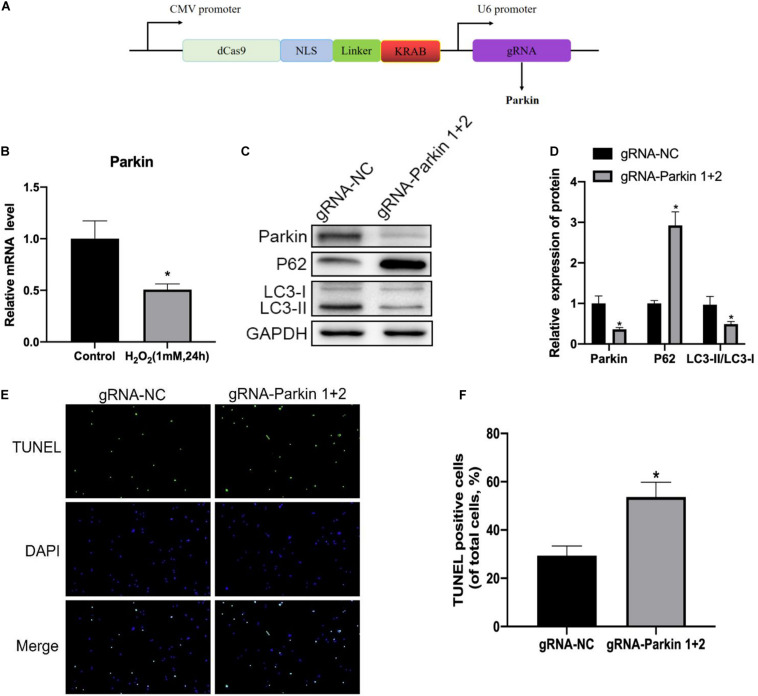
Inhibition of Parkin by the dCas9-KRAB system significantly attenuates H_2_O_2_-induced mitophagy. **(A)** Vector map of clustered regularly interspaced palindromic repeat epigenome editing vectors targeting the Parkin gene promotor region. **(B)** Expression level of Parkin in NP cells. **(C,D)** Western blot of Parkin, P62, LC3-I, and LC3-II expression in NP cells after Parkin inhibition. **(E,F)** Representative images of TUNEL staining and quantitative analysis showing NP cells apoptosis after Parkin inhibition. *p < 0.05 vs. control group.

In order to investigate the relationship between Parkin and IDD in rat NP cells, we determined Parkin expression under oxidative stress by Western blotting, and we found that the Parkin expression decreased significantly in the H_2_O_2_ group compared with that in the control group (Figure 6B). To further verify the role of Parkin in mitophagy, we used the CRISPR/dCas9-KRAB system to repress the expression of Parkin (Figure 6A). We found that mitophagy decreased markedly (Figures 6C,D) while apoptosis of NP cells increased dramatically (Figures 6E,F) after repression of Parkin mediated with CRISPR/dCas9-KRAB. Overall, these results suggested that the activation of mitophagy against apoptosis was dependent on Parkin.

## Discussion

Intervertebral disk degeneration is a common reason for LBP, which is one of the leading causes of chronic disability. To date, conventional treatments including physical therapy, anti-inflammatory medications, and surgeries remain unsatisfactory (Legrand et al., 2007). Therefore, development of biological therapies to prevent IDD is an issue that needs to be solved urgently. Studies have shown that excessive apoptosis of NP cells plays a crucial role in the development of IDD (Guo et al., 2018; Yu et al., 2018). In addition, previous studies have shown that oxidative stress participates in the apoptosis process of NP cells (Nan et al., 2020). Nevertheless, the underlying mechanisms of apoptosis induced by oxidative stress have not been fully clarified.

**FIGURE 7:**
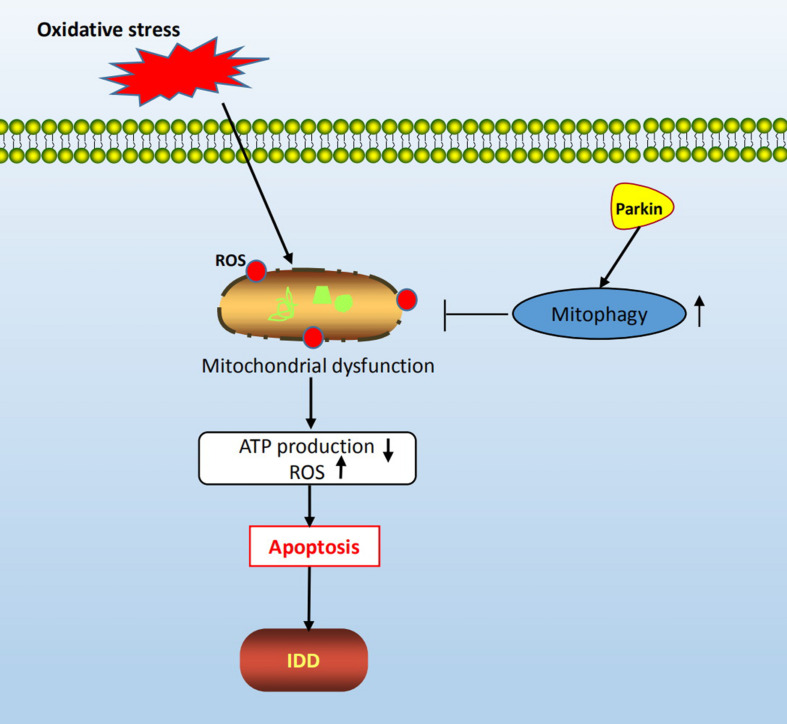
Schematic diagram of mechanisms shows the protective role of mitophagy in rat NP cells under oxidative stress.

In this study, we confirmed that both apoptosis and autophagy were involved in the pathogenesis of oxidative stress-induced IDD. In addition, we found that oxidative stress resulted in mitochondrial dysfunction, autophagy inhibition, and a significant increase in the rate of apoptosis of NP cells. Furthermore, we investigated the relationship between autophagy and apoptosis under oxidative stress by repressing autophagy and found that apoptosis of NP cells was dramatically enhanced, indicating that autophagy played a protective role against apoptosis in response to oxidative stress. Finally, mechanism study revealed that the expression of Parkin was suppressed after H_2_0_2_ treatment, and downregulation of Parkin by dCas9-KRAB led to aggravation of NP cell apoptosis and autophagy inhibition. Hence, we propose that the activation of Parkin could prevent oxidative stress-induced apoptosis and mitochondrial dysfunction in rat NP cells *via* the promotion of mitophagy (Figure 7).

Apoptosis of NP cells is thought to play a critical role in the progression of IDD. In mammals, there are two main apoptotic pathways: the extrinsic (also called death receptor) and the intrinsic (also called mitochondrial) (Wajant, 2002). It is acknowledged that oxidative stress-induced mitochondrial dysfunction is one of the important mechanisms that trigger the intrinsic pathway of apoptosis in NP cells (Lin et al., 2020). An oxidative injury leads to decrease in mitochondrial membrane potential and ATP production, and increase in mitochondrial outer membrane permeability, followed by the release of cytochrome c and activation of caspase cascade, which eventually leads to apoptosis (Chen et al., 2003). Exposure to hydrogen peroxide (1 mmol/L for 24 h) elicited oxidative stress, and we confirmed that both mitochondria injury and apoptosis of NP cells increased markedly after H_2_O_2_ treatment. The results show that oxidative stress causes mitochondrial-mediated intrinsic apoptosis of NP cells. Therefore, maintenance of mitochondrial function and structural integrity is the key to prevent NP cell apoptosis and IDD development.

In recent years, mitophagy has attracted increasing research interest because of its important role in the clearance of damaged mitochondria. Numerous research studies have shown that proper activation of mitophagy plays a cytoprotective role against apoptosis while excessive mitophagy leads to apoptosis (Park and Park, 2013; Lan et al., 2021). A great effort has been made to clarify the relationship between mitophagy and apoptosis, however, the role of mitophagy in ROS-induced apoptosis of NP cells remains unclear. In this study, we used 3-MA to inhibit mitophagy and found that the apoptosis rate of NP cells increased significantly, indicating that mitophagy might be the cellular self-defense mechanism for oxidative stress damage. The results are consistent with those of some previous studies (Wang J. et al., 2018; Wang Y. et al., 2018). For example, the study conducted by Lin et al. (2020) showed that mitophagy suppressed NP cells apoptosis under oxidative stress and attenuated IDD. However, opposite conclusions have also been reported in other studies. For example, Zhan et al. (2020) found that autophagy activation could promote NP cell apoptosis, senescence, and ECM catabolism. Nevertheless, more and more studies show that basal-level autophagy plays a housekeeping and cytoprotective role; while autophagy dysfunction, either inadequate or excessive autophagy, leads to the death of cells. In addition, the pro-survival or pro-death effect of autophagy is also attributed to cell types, time, and degree of stimulus.

Parkin-mediated mitochondrial autophagy is the most intensively investigated mitochondrial autophagy pathway. The autophagy mechanism is translocation of Parkin to defective mitochondria and recruitment of p62/SQSTM1, followed by engulfment of damaged mitochondria by autophagosomes, and degradation by lysosomes (Nguyen et al., 2016). Parkin, as a protective protein, plays many beneficial roles in preventing degenerative diseases, such as Parkinson’s disease (Kamienieva et al., 2021), osteoarthritis (Ansari et al., 2018), and Alzheimer’s disease (Zhao et al., 2021). However, studies regarding the relationship between Parkin and IDD are limited. Our results revealed that Parkin was downregulated in NP cells under oxidative stress. Moreover, the inhibition of Parkin aggravated NP cell apoptosis, exacerbated mitochondrial dysfunction, and suppressed mitophagy activity, indicating that Parkin was able to prevent NP cell apoptosis and protect mitochondrial function through upregulation of mitophagy. Therefore, Parkin is a promising therapeutic target for the treatment of IDD in the future. As mentioned, there are many conventional methods to repress Parkin gene expression. In this study, we innovatively used the dCas9-KRAB system to inhibit the Parkin gene expression in NP cells under oxidative stress. The CRISPR/Cas9 system, which is derived from *Streptococcus pyogenes*, is a powerful genetic editing tool and is widely used in mammalian cells (Ran et al., 2013). CRISPR-dCas9-KRAB is a new tool to silence a target gene expression with high efficiency, low cost, and easy operation. However, the application of the CRISPR/Cas9 system in the intervertebral disk field is limited. A previous study by Stover et al. (2019) explored the potential application of CRISPR epigenome editing to target inflammatory receptors for pain modulation in degenerative intervertebral disk. Our study confirmed that the potential application of CRISPR/Cas9 as a novel gene therapy tool for IDD.

This study has several limitations. First, although our results suggest that Parkin could be a potential therapeutic target for IDD, this study is mainly *in vitro*; thus, further *in vivo* studies are needed to be carried out to provide more convincing evidence. Second, in this study we used NP cells from rats instead of NP cells from humans, and species may cause some differences. Third, since both AF cells and CEP chondrocytes also take part in the degeneration process, the response of AF cells and CEP cells to oxidative stress needs to be elucidated in the future.

## Conclusion

In summary, this study has demonstrated that Parkin is involved in the pathogenesis of IDD and that it plays an important role in the clearance of damaged mitochondria *via* modulation of mitophagy. These findings suggest a potential therapeutic application of Parkin for the prevention and treatment of disk degeneration.

## Data Availability Statement

The raw data supporting the conclusions of this article will be made available by the authors, without undue reservation.

## Ethics Statement

The animal study was reviewed and approved by The Animal Care and Use Committee of the Shenzhen Second People’s Hospital.

## Author Contributions

ZS and BY conceived and designed the study. TL, YZ, and N-DL performed the experiments and data analysis. X-SC and TL wrote and revised the manuscript. All authors contributed to the article and approved the submitted version.

## Conflict of Interest

The authors declare that the research was conducted in the absence of any commercial or financial relationships that could be construed as a potential conflict of interest.
